# Universal behavior of the apparent fragility in ultraslow glass forming systems

**DOI:** 10.1038/s41598-019-42927-y

**Published:** 2019-05-02

**Authors:** Aleksandra Drozd-Rzoska

**Affiliations:** Institute of High Pressure Physics Polish Academy of Sciences, ul. Sokołowska 29/37, 01-142 Warsaw, Poland

**Keywords:** Phase transitions and critical phenomena, Phase transitions and critical phenomena

## Abstract

Despite decades of studies on the grand problem of the glass transition the question of well-defined universal patterns, including the key problem of the previtreous behavior of the primary (structural) relaxation time, remains elusive. This report shows the universal previtreous behavior of the apparent fragility, i.e. the steepness index *m*_*P*_ (*T* > *T*_*g*_) = *d log*_*10*_τ(*T*)/*d*( *T*_*g*_/*T*). It is evidenced that *m*_*P*_(*T*) = 1(*T* − T*), for *T* → *T*_*g*_ and  T*= *T*_*g*_ − Δ T*. Basing on this finding, the new 3-parameter dependence for portraying the previtreous behavior of the primary relaxation time has been derived: *τ*(*T*) = *C*_Ω_((*T* − T*)/*T*)^−Ω^ × [*exp*((*T* − T*)/*T*)]^Ω^. The universality of obtained relations is evidenced for glass formers belonging to low molecular weight liquids, polymers (melt and solid), plastic crystals, liquid crystals, resins and relaxors. They exhibit clear preferences either for the VFT or for the critical-like descriptions, if recalled already used modeling. The novel relation can obey even above the dynamic crossover temperature, with the power exponent Ω ranging between ~17 (liquid crystals) to ~57 (glycerol), what may indicate the impact of symmetry on the previtreous effect. Finally, the emerging similarity to the behavior in the isotropic phase of nematic liquid crystals is recalled.

## Introduction

The glass transition has remained the grand challenge of solid state physics and material engineering since decades. Its most attractive feature constitutes universal previtreous patterns for dynamic properties shared amongst variety of glass forming systems, ranging from low molecular weight liquids to resins, polymers, liquid crystals, plastic crystals, …, and even biosystems^1–9^. The common key feature is the super-Arrhenius (SA) evolution of the primary (structural) relaxation time *τ(T*), viscosity *η(T)*, ..^8,9^:

1 $$\tau (T)={\tau }_{0}\exp (\frac{{E}_{a}(T)}{RT})$$

where *T* > *T*_*g*_, *T*_*g*_ means the glass temperature; *E*_*a*_*(T)* denotes the apparent activation energy: for *E*_*a*_*(T)* = *E*_*a*_ = *const* the basic Arrhenius dependence is obtained. The parallel relation obeys for viscosity.

Angell *et al*.^10,11^ proposed normalized plots log_10_*η*(*T*) and log_10_*τ*(*T*) versus *T*_*g*_/*T* <1, *τ*(*T*_*g*_) = 100*s* and *η*(*T*_*g*_) = 10^13^*Poise*, enabling the common presentation of the SA previtreous behavior for different glass formers. For the “Angell plot’ the simple Arrhenius behavior appears as the straight line and the SA one via bend curves^8–11^. As the metric of the SA dynamics the slope $m={[d{\mathrm{log}}_{10}\eta (T)/d({T}_{g}/T)]}_{T={T}_{g}}$ or equivalently $m={[d{\mathrm{log}}_{10}\tau (T)/d({T}_{g}/T)]}_{T={T}_{g}}$ called ‘fragility’ was introduced^9,10^. The analysis of variety of glass formers, mainly low molecular liquids and polymers, led to the conclusion that glassformers can be arranged in two categories^8–11^: (i) strong (*m* < 30) with dynamics close to the Arrhenius pattern (*m* ~ 16) and (ii) fragile with the notably SA dynamics (*m* > 30)^8,9,11^. To describe the degree of the SA behavior beyond *T*_*g*_ the steepness index, which can be considered as the apparent fragility metric, was introduced^8,9^:

2 $${m}_{P}={m}_{p}(T)=\frac{d{\mathrm{log}}_{10}\tau (T)}{d({T}_{g}/T)}$$

where T > Tg and the subscript ‘_*P*_’ indicates the isobaric character of m(*T*) changes. There is some ambiguity regarding the steepness index/apparent fragility. In the past it was often written as *m*_*T*_, to stress the temperature dependence. Nowadays, the temperature dependent steepness index is denoted as *m*_*P*_ or *m*_*P*_(*T*) to indicate the isobaric nature; Note that *m*_*p*_(*T*_g_) = *m*.

The premonition of a hidden universality caused that the ‘Angell plot’ has become the symbol of the glass transition physics mystery^1–9^. Studies of the fragility concept have become the central area of the glass transition physics^1,6,8,9^. Nowadays, there is a set of model-relations developed for describing *τ(T)* or *η(T)* previtreous behavior^8,9^ but the most popular remains the Vogel-Fulcher-Tammann (VFT) equation^8,9,12–14^. It was introduced empirically^12–14^, but in subsequent decades it also served as the reference checkpoint for significant glass transition models: the free volume approach^15,16^, Adam-Gibbs entropic model^17,18^, ‘critical’ Tanaka model^3,19^ or the random first-order transition (RFOT) model^1,5,20^. Recent theoretical and numerical studies have uncovered some unity in understanding of glass-forming materials from perspectives of different models, earlier considered as distinct. This was associated with finding quantitative relations between emergent elasticity, the average local volume, and the growth of collective motion in supercooling liquids^16,21,22^. Molecular dynamics insight highlighted the correlation of structural relaxation and vibrational dynamics also for these alternative routes^23^. These studies showed that the previtreous dynamics on approaching the glass transition can equally well describe the relaxation data in the ultraviscous region, mainly via the VFT output relation^16,21–23^. Its nowadays form^8,9^:

3 $$\tau (T)={\tau }_{0}{\exp }(\frac{{D}_{T}{T}_{0}}{T-{T}_{0}})$$

where *T* > *T*_g_ and *T*_0_ < *T*_g_ is the VFT singular temperature; *D*_*T*_ denotes the fragility strength coefficient linked to fragility: $m={m}_{\min }+\mathrm{ln}\,10{m}_{\min }/{D}_{T}$, ${m}_{\min }={\mathrm{log}}_{10}\tau ({T}_{g})-{\mathrm{log}}_{10}{\tau }_{0}$^11^. For the prefactor ${\tau }_{0}={10}^{-14}s$ is heuristically assumed^8,9,24^, although the evidence for various glass formers indicates 10^−16^_*S*_ < *τ*_0_ <10^−11^*s*^24–30^.

Despite the success of the VFT portrayal, the clear evidence questioning its fundamental validity exists:

(i). The VFT singular temperature *T*_0_ is linked to the ideal glass (Kauzmann) temperature *T*_*K*_^8,27,28^. The latter is determined from the heat capacity and structural entropy analysis^8,28^. The empirical coincidence between *T*_0_ and *T*_*K*_ constitutes the key argument for the fundamental significance of VFT relation. However, the state-of-the-art analysis of experimental data shows that 0.8 <*T*_0_/*T*_*K*_ < 2.2, i.e. ${T}_{0}\approx {T}_{K}$ only for selected systems^28^.
(ii). The state-of-the-art square-mean-root analysis of few popular molecular glassformers proved the prevalence of other than VFT descriptions^29–31^.
(iii). The apparent activation energy index ${I}_{DO}=-d\,\mathrm{ln}\,{E}_{a}(T)/d\,\mathrm{ln}\,T$ analysis showed notable limitations of the VFT description^24,32,33^.
(iv). The linearized derivative-based analysis clearly showed the prevalence of the critical-like portrayal for liquid crystals, plastic crystals and also polystyrene, xylitol, …^25,26,34,35^

The latter is associated with the relation^26,34,35^:

4 $$\tau (T)={\tau }_{0}{(\xi (T))}^{z}={\tau }_{0}^{C}{(T-{T}_{C})}^{-\varphi =-z\nu }$$

where *z* is the dynamic exponent and $\nu$ is the power exponent for the correlation length of hypothetical fluctuations-heterogeneities.

Such dependence was first proposed for glass forming liquids and polymers by Souletie and Bernard^36^, recalling heuristically spin glasses dynamics. However, the test of results presented in ref.^36^ reveals notable distortions in the immediate vicinity of *T*_g_. Colby^37^ developed the cluster model for polymeric and low-molecular-weight glassformers also leading to eq. (4), with the hypothetically universal exponent *ϕ* = *zv* = 6 × 3/2 = 9. However, the re-analysis of key experimental data from ref.^37^ questioned the suggested description^8,29^. Notwithstanding, the prevalence of eq. (4) for orientationally disordered crystals (ODIC) and liquid crystals (LC) glassformers has been shown^34,35^. Problems of the VFT portrayal led to the appearance of relations avoiding the final temperature singularity, such as Avramov-Milchev (AM)^38^, Elmatad-Chandler-Garrahan (ECG)^39^, and Mauro-Yue-Ellison-Gupta-Allan (MYEGA)^40^, Kivelson-Tarjus-Zheng-Kivelon (KTZK)^41^, Schmidtke-Petzold-Kahlau-Hofmann-Rössler (SPKHR)^42^, … dependences. On the other hand the fundamental validity of the most popular AM^38^ or MYEGA^40^ relations was not supported by the apparent activation energy index analysis^32,33,43^.

The above resume is related to the ultraviscous (ultraslowed) domain near *T*_*g*_. When extending the range of temperatures the dynamic crossover, most often at *T*_*B*_ ~ 1.2*T*_*g*_ − 1.4*T*_*g*_, occurs^8,9^. It is associated with the shift from the non-ergodic (*T* < *T*_*B*_) to the ergodic (*T* > *T*_*B*_) domain^8,9^. The key role in the detection of *T*_*B*_ plays the analysis proposed by Stickel *et al*.^44,45^ via the plot Φ_*T*_(*T*) = *d*log_10_*τ*(*T*)/*d*(1/*T*) vs. 1/*T*. Such analysis results from the comparison of eq. (1) and the VFT eq. (3)^25^:

5 $${[\frac{d\mathrm{ln}\tau (T)}{d(1/T)}]}^{-1/2}={({D}_{T}{T}_{0})}^{-1/2}-\frac{{T}_{0}{({D}_{T}{T}_{0})}^{-1/2}}{T}=A+\frac{B}{T}$$

Recalling eq. (1) one can show that $${H}_{a}(T)=\,\mathrm{ln}\,\tau (T)/(1/T)=({T}_{g}/{\mathrm{log}}_{10}e)[d{\mathrm{log}}_{10}\tau (T)/d({T}_{g}/T)]=({T}_{g}/{\mathrm{log}}_{10}e)\times $$$${m}_{P}(T)$$, where *H*_*a*_(*T*) denotes the apparent activation enthalpy. Then, one can consider [*H*_*a*_(*T*)]^−1/2^ and $${[{m}_{P}(T)]}^{-1/2}$$ vs. $$1/T$$ plots^25^ as parallels of the Stickel *et al*.^44,45^ analysis. Using such analysis^44,45^ and its pressure counterpart^46^ the pressure-temperature invariance of the dynamical crossover time scale $$\tau ({T}_{B},{P}_{B})$$ for the given glass former was suggested^47^. Novikov and Sokolov^48^, using $${{\rm{\Phi }}}_{T}(1/T)$$ plots for 28 glassformers, suggested the ‘magic, universal’ time-scale $$\tau ({T}_{B})={10}^{-7\pm 1}$$ s. However, this concept was criticized due to the large discrepancies for few systems^8,49,50^. It is notable that for the general scaling pattern for describing dynamics in the high temperature domain the relation resulted from the Mode-Coupling-Theory (MCT) is suggested^51^:

6 $$\tau (T)={\tau }_{0}^{MCT}{(T-{T}_{C})}^{-\phi }$$

The experimental evidence, based on ${\tau }^{1/\phi }$ vs. $T$ plot, showed that eq. (6) can obey for $T > {T}_{C}+10K$, ${T}_{C}\approx {T}_{B}$, usually with $1.3 < \phi  < 3.5$^8,9,49,52,53^.

This report presents the universal, previtreous anomaly of the apparent fragility ${m}_{P}(T)$. It is the base for deriving the new equation for portraying the previtreous behavior of $\tau (T)$. Subsequently, novel conclusions regarding the dynamic crossover phenomenon are presented.

## Methods

Results presented are the consequence of the analysis of the primary (structural) relaxation time basing on the high-resolution broadband dielectric spectroscopy (BDS) studies. They were carried out for supercooled system ranging from low molecular weight liquids (glycerol)^54^, polymers (polystyrene, polyvinylidene disulfide – PVDF)^55^, resin (EPON 828: diglycyl ether of bisphenol-A)^56^, liquid crystals (5CB: pentylcyanobiphenyl)^57^, 8*OCB (iso-octyloxycyanobiphenyl)^58^, disordered orientational crystals (ODIC, plastic crystals: the mixture of neopentyl alcohol (NPA) and neopentylglycol (NPG))^59^ and the relaxor glass former in a hybrid system (30% volume fraction of 2 *μm* BaTiO_3_ microparticles in PVDF)^55^. Regarding liquid crystalline glassformers: 5CB exhibits the Isotropic – Nematic (I-N) transition at ${T}_{I-N}\approx 304K$ and crystallizes at ${T}_{cryst.}\approx 290K$^57^.

These studies are supplemented by the extended temperature range tests in the series of polyalcohols: glycerol, threitol and sorbitol^60^ focusing also on the dynamical crossover phenomenon^44,45^. The careful degassing of samples and the proper design of the measurement capacitor enabled the supercooling in the nematic phase down to the glass transition LC glassformers^57^. 8*OCB can be supercooled to ${T}_{g}$ in the isotropic phase^58^. Numerical values of results related to the analysis of glassy dynamics in these materials are given in Tables 1–4 in the Supplementary Information. Results presented were obtained using Novocontrol Broad Band Impedance Analyzer, model 2015. The flat-parallel, gold- coated, measurement capacitor with the bulk gap $d=0.2mm$ and ${U}_{meas.}=1V$ was placed in the Quattro temperature control unit. The relaxation time was determined from the peak frequency of primary relaxation process loss curves as $\tau =1/2\pi {f}_{peak}$^8^.

## Results and Discussion

**Figure 1:**
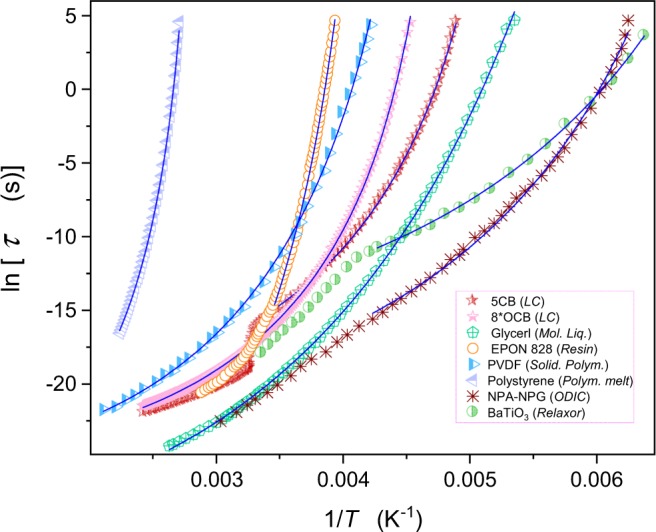
Temperature dependences of the previtreous behavior for tested glass formers: Solid curves are related to eq. (9), with parameters given in Table 1 (Suppl. Info.).

Experimental $\tau (T)$ dependences for eight tested glass formers in the ultraviscous domain are presented in Fig. 1.

**Table 1:** Values of the dynamical crossover temperatures, ${T}_{B}$ resulted from the Stickel *et al*.^45,46^ (*VFT focused*), ‘critical-like’ and $1/{m}_{P}(T)$ analysis.

Glass former	‘Stickel *et al*.’ (K) ${{\boldsymbol{T}}}_{{\boldsymbol{B}}}$	‘critical-like’ (K) ${{\boldsymbol{T}}}_{{\boldsymbol{B}}}$	${\bf{1}}/{{\bf{m}}}_{{\bf{P}}}({\bf{T}})$ (K) ${T}_{{\boldsymbol{B}}}$
glycerol	260	*absence*	295
threitol	330	310	320
sorbitol	*absence*	335	340

Table 1 shows the notable scatter of ${T}_{B}$ values obtained by different methods, which has to be matched with the scatter of the time-scale $\tau ({T}_{B})$. In the opinion of the author, this also indicates one of the possible reasons of problems associated with the ‘magic time scale’ $\tau ({T}_{B})$ scatter.

Notable is the manifestation of the isotropic-nematic (I-N) transition in 5CB at ${T}_{I-N}\approx 304K$^57^. For the ‘hybrid’ relaxor glassformer the SA dynamics terminates at 244 K, where the crossover to the Arrhenius behavior occurs^55^. The plastic crystal NPA-NPG melts at ${T}_{m}\approx 240K$^59^. For each set of experimental data the lowest measured temperature corresponds to $\sim {T}_{g}$.

**Figure 2:**
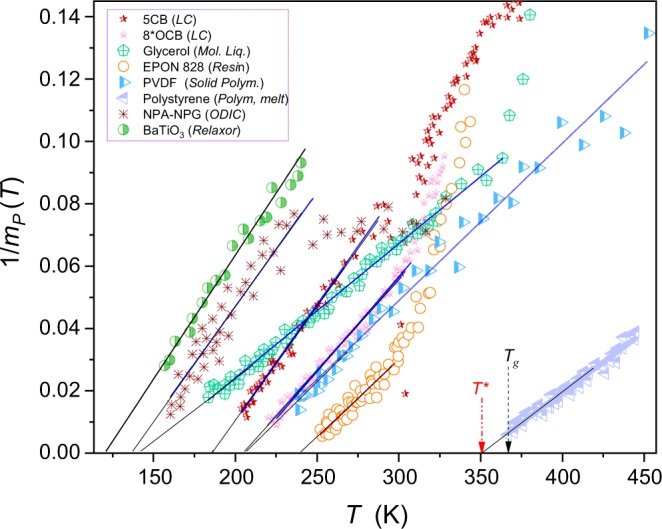
Temperature dependences of reciprocals of apparent fragilities for experimental data from Fig. 1. Arrows indicate the glass temperature *T*_*g*_ and the extrapolated ‘pseudospinodal’ singular temperature T^*^.

Figure 2 presents the evolution of apparent fragilities for data from Fig. 1, calculated via eq. (2). The applied scale reveals the previtreous anomaly:

7 $$\frac{1}{{m}_{P}(T)}=aT+b\to {m}_{P}(T)=\frac{{a}^{-1}}{T-{T}_{g}^{\ast }}$$

where ${T}_{g}^{\ast }={T}_{g}-\Delta {T}_{g}^{\ast }$ and $\Delta {T}_{g}^{\ast }$ can be proposed as the measure of the ‘discontinuity’ of the glass transition; the singular temperature is determined as $1/{m}_{P}({T}_{g}^{\ast })=0$.

The same form the apparent fragility ‘anomaly’ occurs for all tested glass formers. Linking eqs (2) and (7) one obtains:

8 $${m}_{P}(T)=\frac{d{lo}{{g}}_{10}\tau (T)}{d({T}_{g}/T)}=\frac{a}{T-{T}_{g}^{\ast }}$$

The multiplication by $d({T}_{g}/T)$ and the subsequent integration yields the new relation for $\tau (T)$ previtreous evolution:

9 $$\tau (T)={C}_{{\rm{\Omega }}}{(\frac{T-{T}_{g}^{\ast }}{T})}^{-{\rm{\Omega }}}{[{\exp }(\frac{T-{T}_{g}^{\ast }}{T})]}^{{\rm{\Omega }}}={C}_{{\rm{\Omega }}}{(\frac{T-{T}_{g}^{\ast }}{T})}^{-{\rm{\Omega }}}{\exp }({\rm{\Omega }}\frac{T-{T}_{g}^{\ast }}{T})$$

where $T > {T}_{g}$, ${C}_{{\rm{\Omega }}}$, and ${\rm{\Omega }}$ are constants.

The applications of the eqs (7) and (9) are graphically shown in Figs 1 and 2, with parameters in Tables 1 and 2 (Suppl. Info.). Notable, that tested systems belong to different types of glass formers, showing the strong preference for the critical – like the description (5CB, ODIC…)^34,35^ or for the VFT one (glycerol, EPON 828, PVDF…)^35,54,55^.

Regarding physical properties behind parameters in eq. (9) one can show that:

10 $${\rm{\Omega }}=\,\mathrm{ln}\,10\times m\times \frac{{T}_{g}/{T}_{g}^{\ast }}{[1/({\rm{\Delta }}{T}_{g}^{\ast }/{T}_{g})-1]}$$

The power exponent depends on the fragility ($m={m}_{P}({T}_{g})$) and relative values of the glass temperature (${T}_{g}/{T}_{g}^{\ast }$) and the glass transition discontinuity ($\Delta {T}_{g}^{\ast }/{T}_{g}$). In the similar way one can derive the relation: ${C}_{{\rm{\Omega }}}={10}^{2{\rm{\Omega }}}(\Delta {T}_{g}^{\ast }/{T}_{g})/{\exp }(\Delta {T}_{g}^{\ast }/{T}_{g})$ for the prefactor. Consequently, all parameters for eq. (9) can be estimated *in prior* from $1/{m}_{P}(T)$ vs. $T$ analysis.

Results presented in Figs 1 and 2 focus on the ultraviscous/ultraslowed domain close to ${T}_{g}$, with the exception of liquid crystalline 5CB and polymeric PVDF where the description via eq. (9) extends up to $T-{T}_{g} \sim 200K$, with only local distortions caused by the phase transition. Generally, the extension of the temperature range beyond the ultraviscous/ultraslowed domain leads to the crossover to the high temperature dynamical domain (see Introduction).

**Figure 3:**
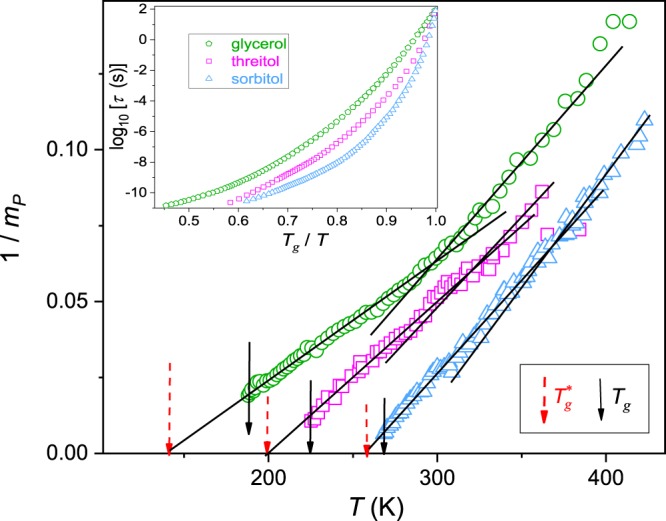
The previtreous behavior of the primary relaxation time and the apparent fragility for glycerol, threitol and sorbitol in the broad range of pressures. The inset presents the relaxation time evolution in the form of the ‘Angell plot’. The main part is for the reciprocal of the apparent fragility. Notable is the manifestation of the previtreous behavior (eqs 7 and 11) in both dynamical domains.

The inset in Fig. 3 presents $\tau (T)$ experimental data for glycerol, threitol and sorbitol, from the homologous series of polyalcohols, in the extended range of temperatures covering both dynamical domains. The main part of Fig. 3 shows the reciprocal of the apparent fragility for these experimental data. It is visible that both dynamical domains are associated with the parallel previtreous behavior of ${m}_{P}(T)$: for $T > {T}_{B}$ described by:

11 $$\frac{1}{{m}_{P}(T)}=a^{\prime} T+b^{\prime} \to {m}_{P}(T)=\frac{{a^{\prime} }^{-1}}{T-{T}_{B}^{\ast }}$$

where ${T}_{B}^{\ast }={T}_{B}-\Delta {T}_{B}^{\ast }$ and $\Delta {T}_{B}^{\ast }$ is the measure of the ‘discontinuity’ linked to the transformation at ${T}_{B}$; the singular temperature:$1/{m}_{P}({T}_{B}^{\ast })=0$.

Using the similar reasoning as for eq. (9) one obtains for $T > {T}_{B}$:

12 $$\tau (T)={C}_{{\rm{\Omega }}}{(\frac{T-{T}_{B}^{\ast }}{T})}^{-{\rm{\Omega }}}{[{\exp }(\frac{T-{T}_{B}^{\ast }}{T})]}^{{\rm{\Omega }}}={C}_{{\rm{\Omega }}}{[\frac{{\exp }((T-{T}_{B}^{\ast })/T)}{(T-{T}_{B}^{\ast })/T}]}^{{\rm{\Omega }}}$$

Following the reasoning associated with eq. (9) one obtains:

13 $${\rm{\Omega }}=\,{\rm{l}}{\rm{n}}\,10\times {m}_{P}({T}_{B})\times \frac{{T}_{B}/{T}_{B}^{\ast }}{[1/({\rm{\Delta }}{T}_{B}^{\ast }/{T}_{B})-1]}\,{\rm{a}}{\rm{n}}{\rm{d}}\,{C}_{{\rm{\Omega }}}=\tau {[({T}_{B})]}^{{\rm{\Omega }}\tau ({T}_{B})}\frac{{\rm{\Delta }}{T}_{B}^{\ast }/{T}_{B}}{\exp ({\rm{\Delta }}{T}_{B}^{\ast }/{T}_{B})}$$

**Figure 4:**
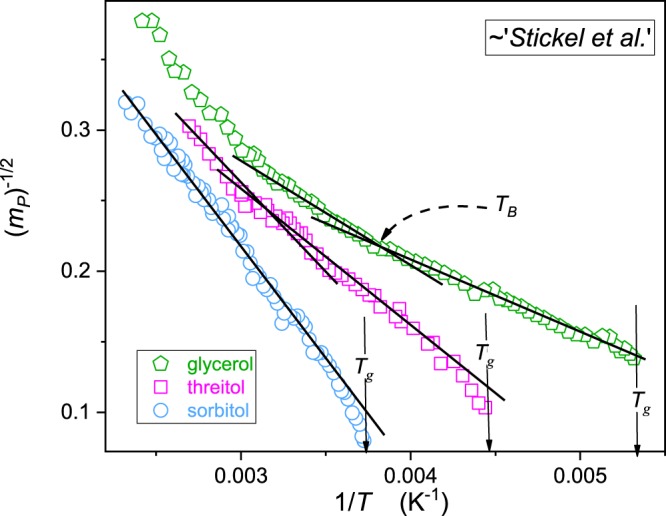
The parallel of Stickel *et al*.^44,45^ plot: *(m*_*P*_*)*^*-1/2*^ ~ *Φ*_*T*_. The straight lines behavior shows the reliability of the VFT portrayal. The intersection of lines estimates *T*_*B*_, dynamic crossover temperature.

When discussing (eqs 9 and 12) for describing $$\tau (T)$$ behavior the question for their comparison with the dominated VFT description arises. For the extended temperature range this has to be also linked to the dynamical domains and the Stickel *et al*.^44,45^ analysis. Figure 4 shows the evolution of $${[{m}_{P}(T)]}^{-1/2}\propto {{\rm{\Phi }}}_{T}=$$$${[d{\mathrm{log}}_{10}\tau (T)/d(1/T)]}^{-1/2}$$ vs. $$1/T$$. As shown in eq. (5) such plot is also the distortions-sensitive test of the validity of the VFT portrayal. Results presented confirms the fair VFT portrayal for glycerol, matched with the estimation of $${T}_{B}$$^8,44,45^. For threitol some discrepancies emerges and for sorbitol there are permanent distortion from VFT description and no reliable estimation of $${T}_{B}$$.

**Figure 5:**
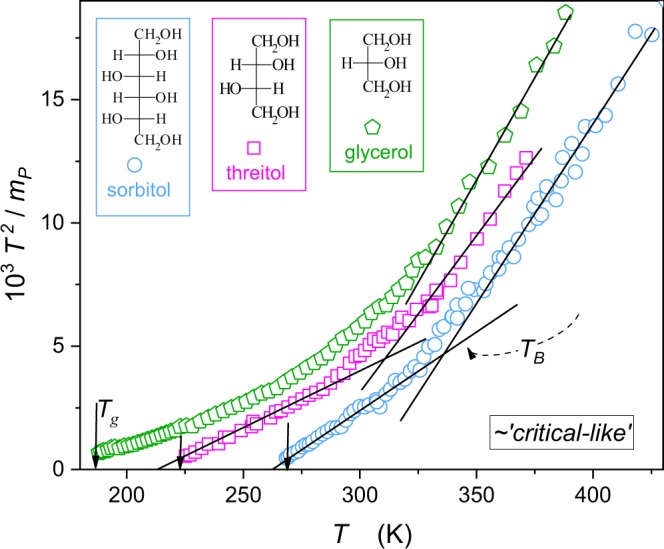
The linearized, derivative-based, plot on the distortions–sensitive validity of critical – like portrayal (eqs 9 and 12). Domains of the possible validity of such description are shown by straight lines. Their intersection can serve as the possible indication of the dynamical crossover temperature. Structures of tested glycerol, threitol and sorbitol are also given.

Figure 5 presents the distortions-sensitive test for the critical-like portrayal, resulting from the comparison of eqs (1) and (4)^25^:

14 $$\frac{{T}^{2}}{{m}_{P}(T)}=\frac{\mathrm{ln}\,10}{{T}_{g}}[\frac{{T}^{\ast }}{\phi }+\frac{1}{\phi }T]=A+BT$$

For plots ${T}^{2}/{m}_{T}(T)$ or equivalently ${T}^{2}/{H}_{a}(T)$ the linear behavior of transformed $\tau (T)$ experimental data indicates the preference for the critical-like portrayal, which can also serve for estimating ${T}_{B}$. In Fig. 5 the structure of tested glass formers is also shown. Notable is the emergence of the uniaxial symmetry in the sequence $glycerol\to threitol\to sorbitol$. This is matched with the shift in the preferable dynamics descriptions: *glycerol* (VFT) $\to$ *sorbitol* (‘critical-like’). These results are shown in Figs 4 and 5 and summarized in Tables 3 and 4 in Supplementary Information.

## Conclusions

This report shows the evidence for the ‘universal’ anomaly of the apparent fragility ${m}_{P}(T)\propto 1/(T-{T}^{\ast })$ for previtreous dynamics, being the base for the model-free derivation of the new relation for $\tau (T)$ portraying (eqs 9 and 12). It exhibits some unique features: (i) it consists of the ‘exponential’ and the ‘critical-like terms, (ii) the relative definition of the distance from the singular temperature $t=(T-{T}^{\ast })/T$. The presented discussion also introduces new characteristics for the previtreous behavior: discontinuities $\Delta {T}_{g}^{\ast }$ and $\Delta {T}_{B}^{\ast }$ for the glass transition and the dynamic crossover and the fragility ${m}_{P}({T}_{B})$. Results presented also show the limitation of the general adequacy of the VFT description, what has to be associated with the question of the general validity of the Stickel *et al*.^44,45^ analysis.

Very recently, basing on the model numerical analysis of the vitrification process, Wang *et al*.^61^ concluded: …*We find that the time scale corresponds to the kinetic fragility of liquids* . *Moreover*, *it leads to scaling collapse of both the structural relaxation time and dynamic heterogeneity for all liquids studied*, *together with a characteristic temperature associated with the same dynamic heterogeneity…* .’. Then, one can consider the apparent fragility in the previtreous domain as the metric of dynamics for dynamical heterogeneities in the ultraslowed domain. Consequently, basing on results of this report one can indicate the similarity between the isotropic phase of nematic liquid crystals^62,63^ and the previtreous domain in glass forming systems^1–9^. For the LC isotropic liquid phase^62,63^: (i) the primary relaxation time for $T > {T}_{I-N}$ is described by eqs (1,3,6) and the emergence of the SA description in this high temperature domain is associated with the impact of prenematic fluctuations-heterogeneities in the isotropic liquid (‘fluidlike’) surrounding, (ii) the dynamics of prenematic fluctuations is described via ${\tau }_{fluct.}\propto 1/(T-{T}^{\ast })$ and $T > {T}_{I-N}={T}^{\ast }+\Delta {T}^{\ast }$ (see eqs 8 and 11); (iii) the impact of prenematic fluctuations is very strong for methods related to the 4-point correlation functions (Kerr effect, nonlinear dielectric spectroscopy,..)^62,63^ but for other methods such as density or dielectric constant can be almost negligible, due to the poor contrast factor between fluctuations and their surroundings^64^; (iv) dynamic heterogeneities in supercooled liquids are related to the nanoscale time/space range and they are associated with no more than few tens of molecules, as shown experiments carried out ca. 10 K above ${T}_{g}$ i.e. $T-{T}^{\ast }\approx 40K$ (glycerol)^8,9^ but in the isotropic phase of 5CB one obtains the same parameters for prenematic fluctuations for $T-{T}^{\ast }\approx 40K$^62,63^. Notwithstanding, there is the basic difference the isotropic-nematic and the glass transition. The glass transition is ‘stretched’ in temperature and time whereas the I-N transition is the well-defined discontinuous phase transition. To comment these basic properties, one can indicate the clear difference between symmetries of prenematic fluctuations (heterogeneities) and their ‘fluidlike’ isotropic surrounding, associated with the similar ‘sharp’ difference between symmetries of neighboring isotropic liquid and nematic phases. This can be not the case of heterogeneities in a supercooled system above ${T}_{g}$: their structure can resemble the amorphous surrounding, but with larger solidity and eventually only weak symmetry distortions. Consequently the border between heterogeneities and their surrounding can be gradual and stretched what finally may lead to the ‘stretched transition’ to the amorphous solid state. The progress in understanding and describing the anomalous previtreous increase of the primary (structural) relaxation time or viscosity is considered as the key to resolving the scientific challenge of the glass transition^1–9^. This report presents the new evidence for the previtreous behavior of the relaxation time and the apparent fragility, offering the new gate for glass transition models.

## Supplementary information

Supplementary Information for the paper ‘ Universal behavior of the apparent fragility in ultraslow glass forming systems’
