# Supplementary material for: Universal behavior of the apparent fragility in ultraslow glass forming systems
Source: Sci Rep. 2019 May 2;9:6816. doi: 10.1038/s41598-019-42927-y (PMC6497650; doi:10.1038/s41598-019-42927-y)
Supplement: Supplementary file 1 — Supplementary Information for the paper ‘ Universal behavior of the apparent fragility in ultraslow glass forming systems’ [file 41598_2019_42927_MOESM1_ESM.pdf]

Supplementary Information

for the paper

‘ *Universal behavior of the apparent fragility in ultraslow glass forming systems*’

by Aleksandra Drozd-Rzoska (IHPP PAS, Warsaw, Poland)

e-mail: agatka.malwinka@gmail.com

Issues:

I. Tables (I, II, III and IV) presenting numerical results related to Figures 1 - 5, with comments (**pages 1 – 4**)

II. Discussion of the definition of distance from the singular temperature (**page 5**)

Issue I

Tables presenting results of analysis

Table I Results of fitting of experimental data for previtreous behavior of the structural relaxation time (Fig. 1) via the new eq. (9): the formula is recalled in the Table. Results are coupled to slid curves in Fig.1.

Relation	$ln\tau(T)=lnC_{\Omega}+\Omega\times\left(1-\left(T^{*}/T\right)\right)-\Omega\times ln\left(1-\left(T^{*}/T\right)\right)$							
	Glass Forming Materials							
	5CB	8*OCB	GLY-CEROL	EPON 828	POLY-STYRENE	PVDF	NPA-NPG	RELA-XOR
Parameters: lnC_{Ω}	-40.4	-41.4	-92	-40.7	-30.0	-49.5	-48.4	-44.0
$T_g[K]$	205	220.7	187.7	253.9	369.6	237.4	160.3	155.0
Ω	18.5	16.6	62	26.1	16.5	24.2	26.0	27.2
$\Delta T_{r.}[K]$	80.7	194.1	179.5	41.1	77.4	217.0	283.7	80.7
$T^{*}[K]$	184.6	206.3	139	233.5	349.0	208.7	134.9	123.0
	$\Delta T_{r.}=\Delta T_{range}=\left(T_r-T_g\right)$, where $T_{r.}$ denotes the terminal fitting temperature							

Table II Results of the linear regression fit for the reciprocal of apparent fragilities $[m_P(T)]^{-1}$ in the ultraslowed previtreous domain close to T_g, for glass formers recalled in Fig. 2.

Relation		$m_p(T)=\frac{1}{aT+b}, \quad aT+b\neq 0$							
		Glass Forming Materials							
		5CB	8*OCB	GLY-CEROL	EPON 828	POLY-STYRENE	PVDF	NPA-NPG	RELA-XOR
characteric temperatures [K]	T_g	205.0	220.7	187.7	253.9	369.6	237.4	160.3	155.0
	$\Delta T_{r.}$	83.2	86.1	176.4	42.3	75.4	215.6	284.1	284.1
	T_g^*	185.7	205.5	141.0	239.0	350.0	207.6	136.0	123.8
	ΔT_g^*	19.3	15.2	46.4	14.9	19.6	29.8	24.3	31.2
	$\Delta T_{r.}=(T_{range}-T_g)$, where T_{range} denotes the terminal temperature of fitting								

Table III Parameters characterizing the previtreous anomalies of the apparent fragility and related new relations for $\tau(T)$ portrayal (eqs. (9) and (13)), for three selected glass formers in the extended range of temperatures.

	Parameters: $T(K)$				
System & dynamical domain	Ω	T_g^*<br>T_B^*	ΔT_g^*<br>ΔT_B^*	$m_P(T_g)$<br>$m_P(T_B)$	T_g<br>T_B
low temp. domain<br>GLYCEROL<br>*high temp. domain*	62<br><br>29	141<br><br>210	47<br><br>85	58<br><br>29	187.7<br><br>295
low temp. domain<br>THREITOL<br>*high temp. domain*	28<br><br>17	201<br><br>230	13<br><br>80	100<br><br>20	224<br><br>310
low temp. domain<br>SORBITOL<br>*high temp. domain*	15<br><br>10	260<br><br>280	8<br><br>70	163<br><br>16	268<br><br>350

Table IV Results of the derivative-based, linearized analysis of $\tau(T)$ experimental data related to Fig. 4, and Fig. 5: i.e. to the critical-like and VFT portrayal. The latter tests also the applicability of the Stickel et al. plot.

type of description	**VFT & 'Stickel et al.' analysis**			**'critical-like' analysis**		
parameters	D_T	T_0 (K)	T_B (K)	T_C (K)	*exponent* ϕ	T_B (K)
**glycerol** – *low dyn. domain*	19	124	**260**	*absence*	*absence*	***absence***
**glycerol** – *high dyn. domain*	3.8	204		279	6.1	
**threitol** – *low dyn. domain*	10	178	**330**	214	15.6	**310**
**threitol** – *high dyn. domain*	4.3	207		290	6.5	
**sorbitol** – *low dyn. domain*	~5	~233	***absence***	263	12.8	**340**
**sorbitol** – *high dyn. domain*	*absence*	*absence*		311	7.5	

Issue II

Critical phenomena, glass transition

and the definition of the distance from the 'critical point'.

It is notable that eq. (12) is associated with the 'relative' definition of the distance from the singular temperature: $\left(T-T^*\right)/T=\left(1-T^*/T\right)$. Worth recalling is the discussion regarding the 'absolute' $T-T_C$ or $\left(T-T_C\right)/T_C$ and 'relative' $\left(T-T_C\right)/T$ definitions of the distance from the singular (critical) temperature in the Physics of Critical Phenomena[a]. Although the 'relative' definition was indicated as fundamentally more optimal[b,c], the 'absolute' one is used in practice. This results from the fact that for critical phenomena the description via the leading power term $\left(T-T_C\right)^{-\alpha}$ (α is an critical exponent) is possible in the immediate vicinity of T_C, in practice for $\left(T-T_C\right)<1K$. Then there is no practical difference between both definitions, in this terminal domain This is not the case of glass forming system, for which eq. (12) can obey even up to $T_R\sim T^*+120K$. It is notable that a similar 'relative' definition of the distance from the singular temperature appears for the 'thermodynamic' previtreous anomaly of the structural entropy $S_C(T)\propto\left(1-T_K/T\right)$ or for its generalized form $S_C(T)\propto\left(1-T_K/T\right)^n$.[d-f]

a) Hohenberg, P. C., and Halperin, B. I. Theory of dynamic critical phenomena, *Rev. Mod. Phys.* **49**, 435 (1975)

b) Sengers, J. V., Bedeaux, D., Mazur, P., Greer, S. C., Behavior of the dielectric constant of fluids near a critical point. *Physica A* **104**, 573-594 (1980).

c) Martinez-Garcia, J. C., Rzoska, S. J., Drozd-Rzoska, A., Martinez-Garcia, J. A universal description of ultraslow glass dynamics. *Nat. Comm.* **4**, 1823(9) (2013).

d) Martinez-Garcia, J. C., Rzoska, S. J., Drozd-Rzoska, A., Martinez-Garcia, J., and Mauro, J. C. Divergent dynamics and the Kauzmann temperature in glass forming systems. *Sci. Rep*. **4**, 5160 (2014).

e) Martinez-Garcia, J. C., Rzoska, S. J., Drozd-Rzoska, A., Starzonek, S., and Mauro, J. C. Fragility and basic process energies in vitrifying system, *Sci. Rep.* **5**, 8314 (2015).
